# Epidemiology of drug-resistant tuberculosis among hospitalized children with tuberculosis in southwest China, 2017–2024

**DOI:** 10.3389/fmicb.2025.1609146

**Published:** 2025-07-02

**Authors:** Dong-Mei Wang, Qi An, Qing Yang, Yi Liao

**Affiliations:** ^1^Department of Science and Education Division, Public Health Clinical Center of Chengdu, Chengdu, China; ^2^Department of Clinical Laboratory Medicine, Chengdu Women's and Children's Central Hospital, School of Medicine, University of Electronic Science and Technology of China, Chengdu, China

**Keywords:** pediatric, drug-resistant tuberculosis, epidemiology, clinical characteristics, southwest China

## Abstract

**Background:**

To describe the demographic and clinical characteristics of pediatric tuberculosis (TB) inpatients diagnosed with resistance to any anti-tuberculosis drug [drug-resistant tuberculosis (DR-TB)] in southwest China.

**Methods:**

Patients aged ≤14 years with clinically diagnosed pediatric TB were recruited from January 2017 to December 2024 at specialty hospitals in southwest China based on either etiology or clinical confirmation. Hospitalization records were extracted for each patient.

**Results:**

Among 2,208 pediatric TB patients, 90 (4.08%) had DR-TB. DR-TB cases had an average age of 10.94 ± 3.52 years, with a male-to-female ratio of 0.76:1. The highest proportion was in the 10–14-year age group (72.2%), and prevalence was significantly higher in girls than boys. By disease type, 13.33% had pulmonary tuberculosis, 5.56% had extrapulmonary tuberculosis (EPTB), and 81.11% had combined TB. The most common form of EPTB was lymph node TB (30.00%), followed by pleural TB (20.71%), abdominal TB (19.29%), and TB meningitis (14.29%). Among the 90 pediatric DR-TB cases, 74.4% were primary patients (with rifampicin-resistant TB and multidrug-resistant TB accounting for 36.7 and 30.0%, respectively). The Tibetan ethnic group had the highest proportion of DR-TB cases (63.3%). Over the 8-year period, most pediatric DR-TB cases were from western Sichuan (including Ganzi, Aba, and Liangshan minority areas), with the highest number in the Ganzi Tibetan Autonomous Prefecture.

**Conclusion:**

Pediatric DR-TB in southwest China predominantly affects older girls, with primary cases representing a high proportion. The western regions of Sichuan bear a relatively high burden. Public health efforts should prioritize awareness, screening, and early diagnosis of pediatric DR-TB in high-risk areas to prevent transmission.

## Introduction

Tuberculosis (TB) is a chronic infectious disease caused by *Mycobacterium tuberculosis* (MTB), which has long been a major challenge in global public health. The emergence of drug-resistant tuberculosis (DR-TB), especially multidrug-resistant tuberculosis (MDR-TB) and extensively drug-resistant tuberculosis (XDR-TB), has exacerbated the challenges in TB prevention and control. Children, as a susceptible population for TB, face particularly high rates of DR-TB. Because their immune systems are not fully developed and diagnosis and treatment present many difficulties, the management of DR-TB in children has become a key challenge in global TB control.

Southwest China (including Yunnan, Sichuan, and Chongqing) is a high-burden region for TB. Its unique geographical environment, economic conditions, and population mobility contribute to a particularly complex TB epidemic. In recent years, although China has achieved remarkable progress in TB control, the burden of DR-TB in southwest China remains severe. The diagnosis rate of DR-TB in children is low, treatment is difficult, and drug options are limited, resulting in cure rates that are far lower than those in adults (Trajman and Schwartzman, 2021; Patankar et al., 2023). At the same time, medical resources in some remote areas of southwest China are scarce, and the TB prevention and control system requires further strengthening, hindering early detection and standardized treatment of pediatric DR-TB (Yin et al., 2025; Dharmapalan and Mane, 2023). Addressing these challenges requires urgent efforts to strengthen DR-TB control in children in southwest China. This article aims to review the current status, epidemiological characteristics, and clinical features of pediatric DR-TB in southwest China, analyze the challenges, and advocate for enhanced research and control strategies to protect child health and advance progress toward ending TB.

## Methods

### Study design and population

**Figure 1:**
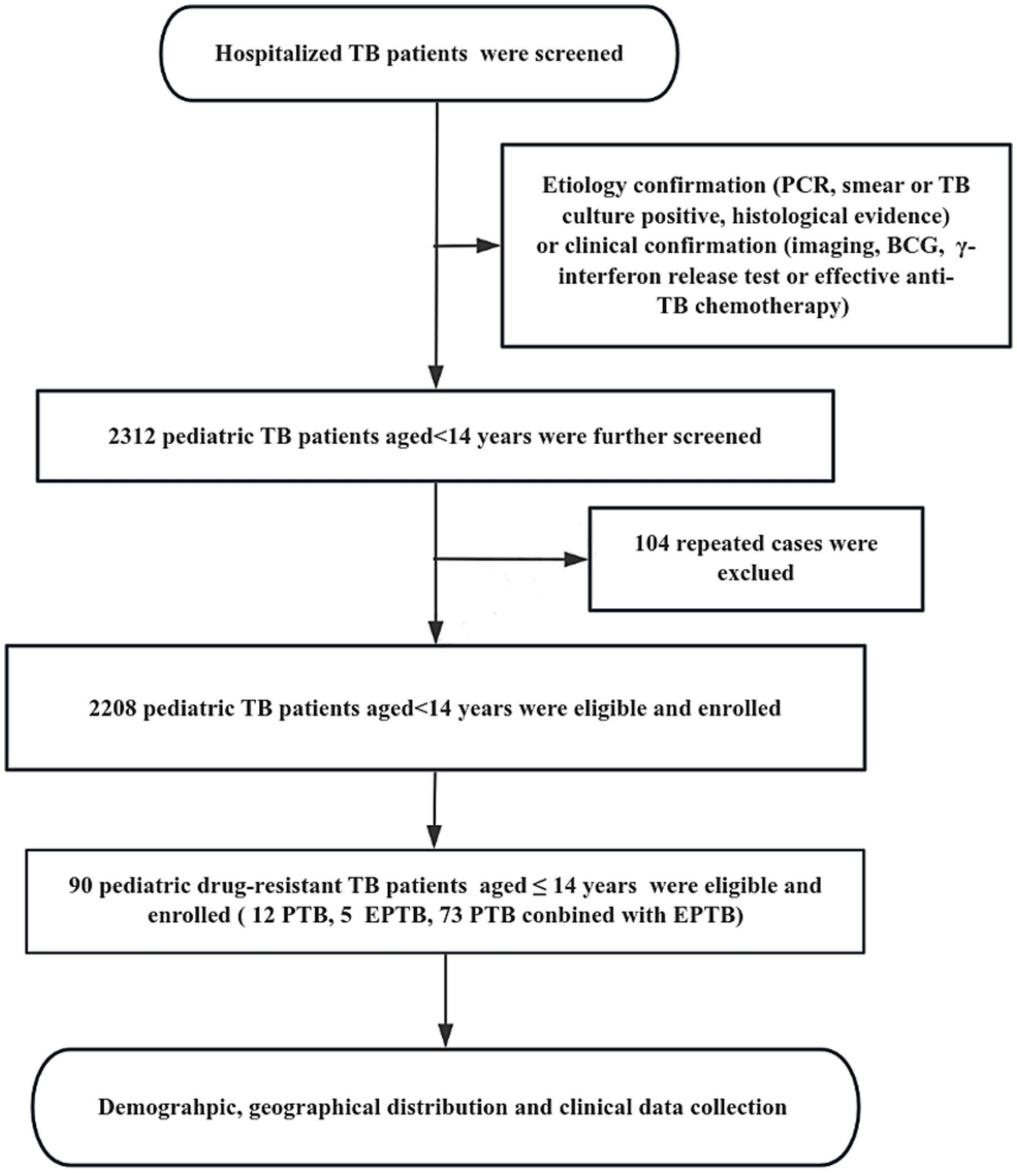
The flow diagram of our study. Demographic information and clinical data was reviewed from the Public Health Clinical Center of Chengdu, Sichuan, China.

This retrospective study was based on discharge data from the Chengdu Public Health Clinical Center (PHCC), located in the central city of Sichuan Province in southwest China. Patients were recruited from January 2017 to December 2024 at PHCC based on either etiology [cases defined as bacteriologically confirmed when biological specimens were positive by smear microscopy, polymerase chain reaction (PCR), or culture, in accordance with World Health Organization (WHO) guidelines] or clinical confirmation [cases without etiological support but diagnosed through imaging findings, Bacillus Calmette-Guérin (BCG) scar assessment, interferon-gamma release assay (IGRA), or response to effective anti-TB chemotherapy]. After eliminating duplicate cases, a total of 2,208 pediatric TB patients aged ≤14 years were included in the study. All information about these cases was obtained from the hospital medical record management system and hospital information system (HIS), including detailed demography, the geographical distribution of habitual residence, clinical characteristics, outcomes, and other relevant information (Figure 1).

### Variables and outcomes

The medical record information of each patient extracted from the HIS included sociodemographic, TB-related and outcome variables. Sociodemographic variables consisted of gender, age, ethnicity, and place of habitual residence. The TB-related variables included TB type, specific diagnosis, complications, and hospital admission time. The outcome variables included treatment outcomes, length of stay in hospital, and TB hospitalization costs. Information about the habitual residence was collected for all cases, then coded according to their residence (using geographic codes), and matched to a 1:100,000 digital map of China using Python 3.7. The cases were displayed using detailed geographical distribution charts of China and Sichuan Province.

### Diagnostic criteria, *Mycobacterium* culture and species identification

Diagnosis of pediatric TB was based on three key standards: the Chinese Pulmonary Tuberculosis Diagnostic Criteria (WS 288–2017), the Chinese TB guidelines for clinical diagnosis and treatment (Chinese Medical Association, 2005), and the updated WHO guidelines (World Health Organization, 2024). The diagnostic workup included smear microscopy, symptom-based clinical evaluation, culture isolation, and molecular testing. Following WHO guidelines, pulmonary tuberculosis (PTB) was defined as TB disease involving the lung parenchyma and/or tracheobronchial tree, including miliary TB cases, with or without extrapulmonary tuberculosis (EPTB), while all other disease sites were classified as EPTB. Concurrent pulmonary and extrapulmonary TB (combined TB) was defined as any TB case involving both lungs and other organs. For mycobacterial culture, we used the BACTEC™ MGIT 960 System. For species identification, MPT64 antigen detection (using colloidal gold immunochromatography), p-nitrobenzoic acid (PNB), and thiophene-2-carboxylic acid hydrazide (TCH) were used to distinguish MTB from *Nontuberculous mycobacteria* (NTM) (Cao et al., 2021). Polymerase chain reaction (PCR) was then used for further species/complex identification following the manufacturer’s instructions (CapitalBio Corp., Chengdu, China) (Wang et al., 2020).

### Drug resistance patterns

Drug sensitivity testing (DST) was performed using both phenotypic (conventional) and genotypic (molecular) methods. The rapid molecular detection methods included Xpert MTB/RIF and fluorescence PCR melting curve analysis, both capable of detecting isoniazid and rifampicin resistance within 1 day. The fluorescence PCR melting curve method using the MeltPro® TB integrated detection system (Zeesan Biotechnology Co., Ltd., Xiamen, China) could identify drug-resistant mutations in rpoB (rifampicin), KatG/inhA/ahpC (isoniazid), rpsL/rrs (streptomycin), embB (ethambutol), and gyrA (fluoroquinolones). Phenotypic DST was performed using the MicroDST™ system (Yinke AUTOBIO Diagnostics Co., Ltd., Zhuhai, China), testing susceptibility to 14 antituberculosis drugs: first-line drugs including isoniazid (0.2–1.6 μg/mL), rifampicin (1–8 μg/mL), ethambutol (2.5–20 μg/mL), and streptomycin (1–8 μg/mL); and second-line drugs including rifapentine (0.5–2 μg/mL), levofloxacin (2–8 μg/mL), amikacin (1–4 μg/mL), capreomycin (2.5–10 μg/mL), protionamide (10–40 μg/mL), pasiniazide (0.5–2 μg/mL), moxifloxacin (0.5–2 μg/mL), p-aminosalicylic acid (2–8 μg/mL), clarithromycin (4–16 μg/mL), rifabutin (0.75–3 μg/mL), kanamycin (2.5–10 μg/mL), and clofazimine (2–8 μg/mL). Quality control was maintained using the H37Rv reference strain (ATCC 25618).

### Laboratory quality control

External quality assessment (EQA) was conducted for smear microscopy, culture, and DST through the Innovation Alliance on Tuberculosis Diagnosis and Treatment (Beijing, China). Additionally, about 10% of isolates were randomly selected for blinded retesting at a superior provincial-level Centers for Disease Control and Prevention laboratory.

### Statistical analysis

All statistical analyses were performed using STATA 17.0 (StataCorp, College Station, TX). Continuous variables with normal distribution were expressed as mean ± standard deviation (SD) and compared using analysis of variance (ANOVA). Non-normally distributed variables were analyzed using nonparametric tests. Categorical variables were presented as frequencies (percentages) and compared using *χ*^2^ tests or Fisher’s exact tests as appropriate. A two-tailed *p* value <0.05 was considered statistically significant.

### Ethics approval and consent to participate

This study protocol was reviewed and approved by the Ethics Committee of PHCC (Approval No. YJ-K2023-08-01). Since all patient data were obtained through routine clinical care and the mandatory national TB reporting system, the requirement for individual informed consent was waived by the ethics committee.

## Results

### Demographic and clinical characteristics

Among the 2,208 pediatric TB patients in this study, 90 cases were diagnosed with pediatric DR-TB. In pediatric DR-TB patients, 43.3% (39/90) were male and 56.7% (51/90) were female, resulting in a male-to-female ratio of 0.76:1. The mean age of the 90 pediatric DR-TB patients was 10.94 ± 3.52 years (range: 279 days to 14 years). Among the patients, 6.7% (6/90) were aged 0–4 years, 21.1% (19/90) were aged 5–9 years, and the highest proportion was in the 10–14 years group (72.2%, 65/90). Among the 90 pediatric DR-TB cases, 74.4% (67/90) were primary patients, and RR-TB and MDR-TB accounted for 36.7% (33/90) and 30.0% (27/90), respectively. The number of retreated cases accounted for 25.6% (23/90) of the total cases, and the MDR-TB in the retreated cases was 16.7% (15/90). Regarding population distribution, the Tibetan ethnic group had the highest proportion of DR-TB cases in children (63.3%, 57/90) (Supplementary Table 1; World Health Organization, 2009).

Out of 90 cases of pediatric DR-TB, 84.4% (76/90) showed abnormal chest x-rays, displaying varying degrees of the following findings: patchy shadows (68.9%, 62/90), nodules (65.6%, 59/90), cavity (21.1%, 19/90), hydrothorax (20.0%, 18/90), and hydropericardium (3.3%, 3/90). The most commonly associated clinical symptoms were cough (54.4%, 49/90), headache (15.6%, 14/90), and vomiting (12.2%, 11/90). The most common co-occurring complications were hyperuricemia (44.4%, 40/90), pulmonary bacterial infection (41.1%, 37/90), and anemia (23.3%, 21/90). Among the 90 children with DR-TB, 94.4% (85/90) were discharged after their condition improved and was controlled, while 5.6% (5/90) of patients requested early discharge for personal reasons (Supplementary Table 1).

### Type distribution and age proportion among PTB, EPTB, and combined TB groups

**Figure 2:**
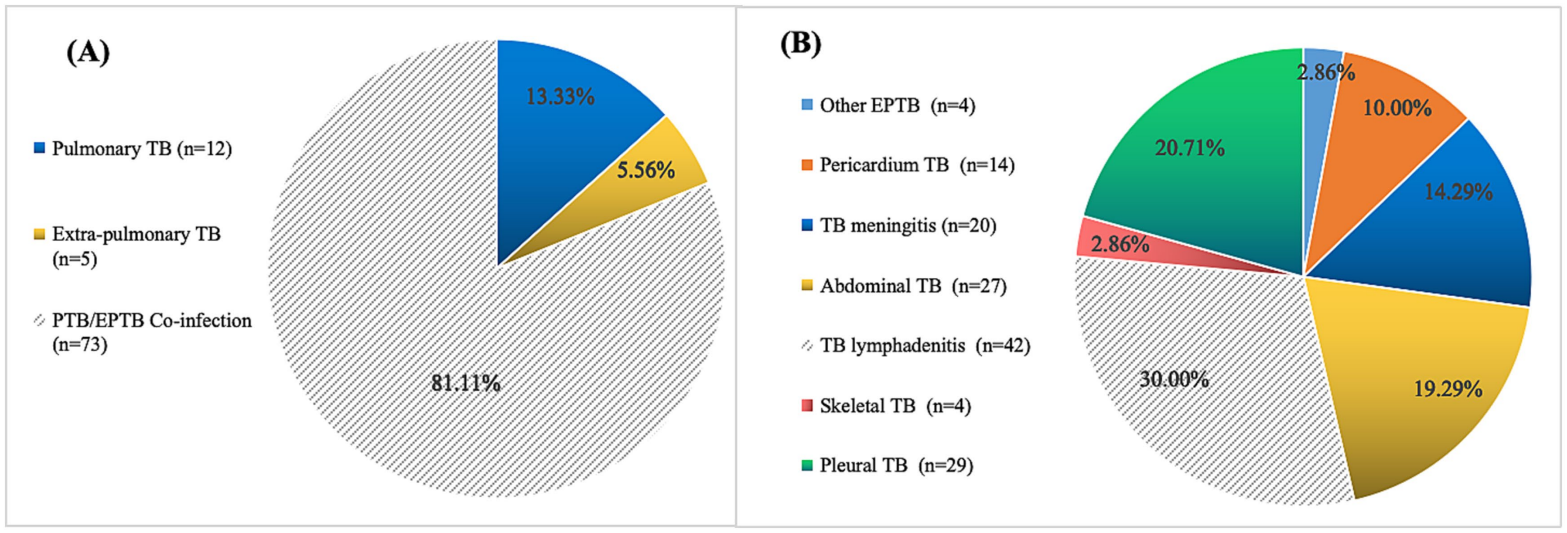
Location and distribution of pediatric drug-resistant TB cases. **(A)** Location and distribution of lesions in 90 pediatric drug-resistant TB cases. **(B)** Tissue type distribution of 78 EPTB and PTB/EPTB co-infection cases (21 cases of disseminated TB involved 2 extrapulmonary lesions, 16 cases at 3 sites and 6 cases at 4 sites).

Among the pediatric DR-TB patients, 13.33% (12/90) had PTB, 5.56% (5/90) had EPTB, and 81.11% (73/90) had combined TB (Figure 2A) (*χ*^2^ = 5.687, *p* = 0.031). The most common form of pediatric DR-TB was TB lymphadenitis (30.00%), followed by pleural TB (20.71%), abdominal TB (19.29%), TB meningitis (14.29%), pericardial TB (10.00%), skeletal TB (2.86%), and other forms of EPTB (2.86%). Cases of disseminated TB involved 2 extrapulmonary lesions in some patients, 16 cases at 3 sites, and 6 cases at 4 sites (Figure 2B).

### Microscopy, culture and gene sequencing results

**Figure 3:**
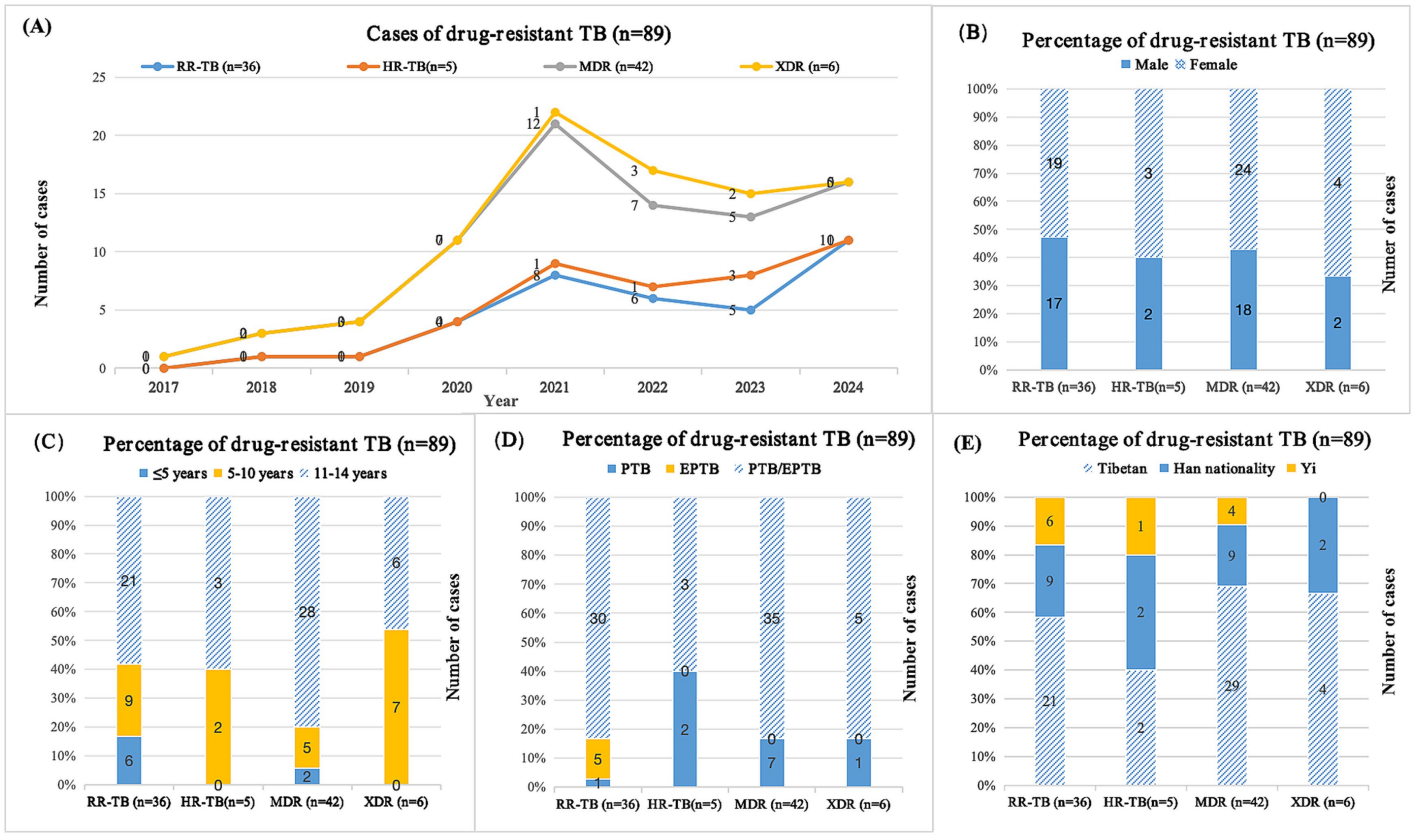
Cases of pediatric drug-resistant TB patients in southwest of China. **(A)** Distribution of cases between different years; **(B)** Distribution of cases in different genders; **(C)** Distribution of cases in different age groups; **(D)** Distribution of cases of different types of tuberculosis; **(E)** Distribution of cases among different ethnic groups; TB, tuberculosis; RR-TB, rifampicin-resistant tuberculosis; HR-TB, isoniazid-resistant tuberculosis; MDR, multidrug-resistant tuberculosis; XDR, extensive drug-resistant tuberculosis; PTB, pulmonary tuberculosis; EPTB, extrapulmonary tuberculosis. Among 90 cases of pediatric drug-resistant tuberculosis, one case was single resistant to fluoroquinolones.

Among the pediatric DR-TB patients in this study, all were diagnosed as positive for the pathogen, and 3 cases were complicated with NTM infection (including 1 case each of *M. lentiflavum*, *M. fortuitum*, and *M. simiae*). DST results of 90 DR-TB cases were confirmed by mycobacterial culture in 33 cases and molecular diagnosis in 57 cases. Except for one case of female urogenital TB with single fluoroquinolone resistance, cases were divided into four drug resistance groups based on DST results: rifampicin-resistant tuberculosis (RR-TB), isoniazid-resistant tuberculosis (HR-TB), MDR-TB, and XDR-TB. The classification and statistical results showed that RR-TB, HR-TB, MDR-TB, and XDR-TB incidence from 2017 to 2024 showed varying degrees of upward trend (Figure 3A). The proportion of DR-TB in girls was significantly higher than that in boys (Figure 3B), with the majority of cases occurring in 11-14-year-old children (Figure 3C). In the distribution of tissue types in DR-TB cases, PTB/EPTB co-infection accounted for the highest proportion (Figure 3D). In terms of ethnic distribution, the proportion of Tibetans in RR-TB, MDR-TB, and XDR-TB groups was significantly higher than that in other groups (Figure 3E).

### Disease incidence trend and geographical distribution

**Figure 4:**
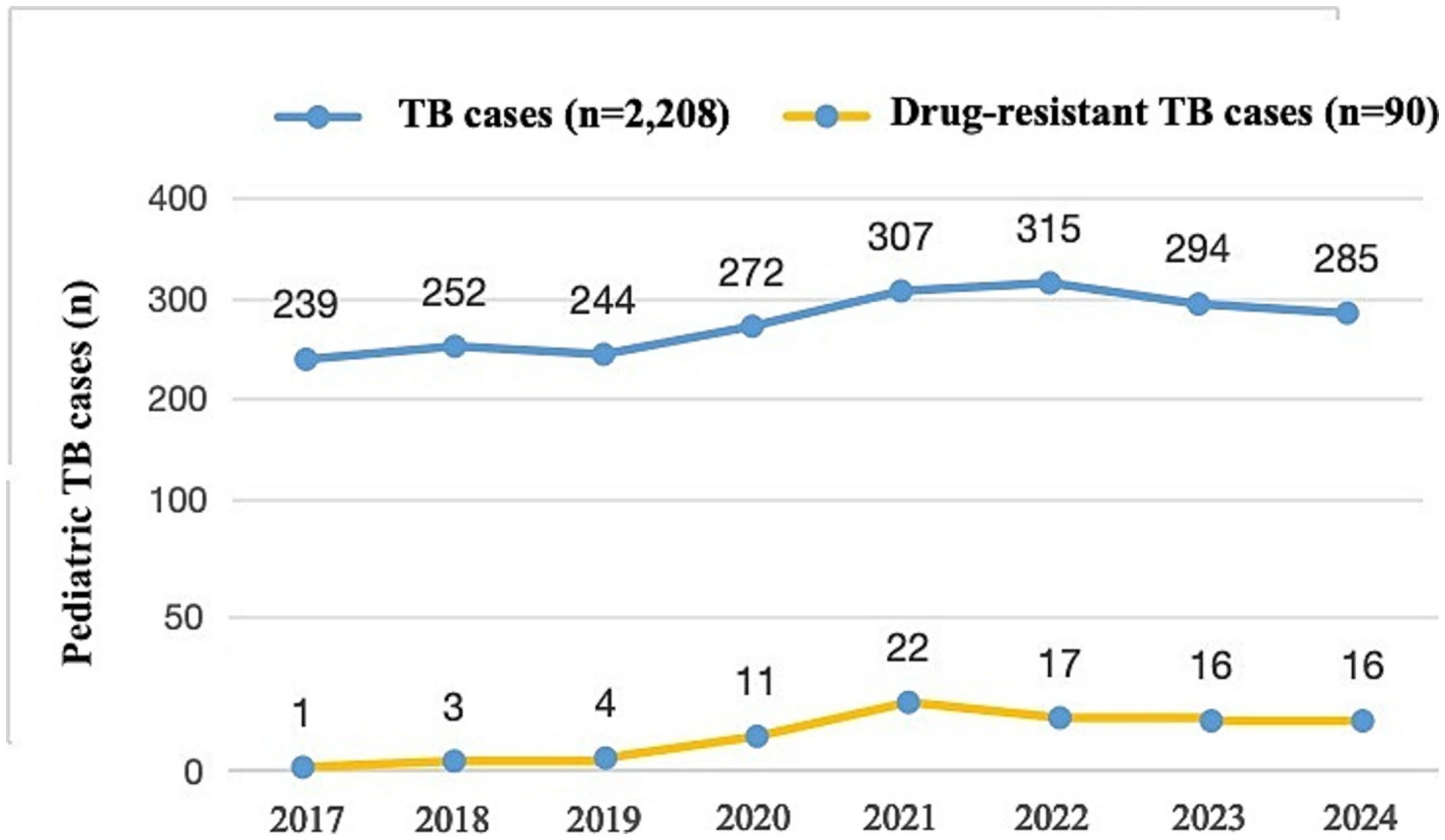
Numbers of pediatric drug-resistant TB patients presenting each year between 2017 and 2024 (*n* = 2,208).

**Figure 5:**
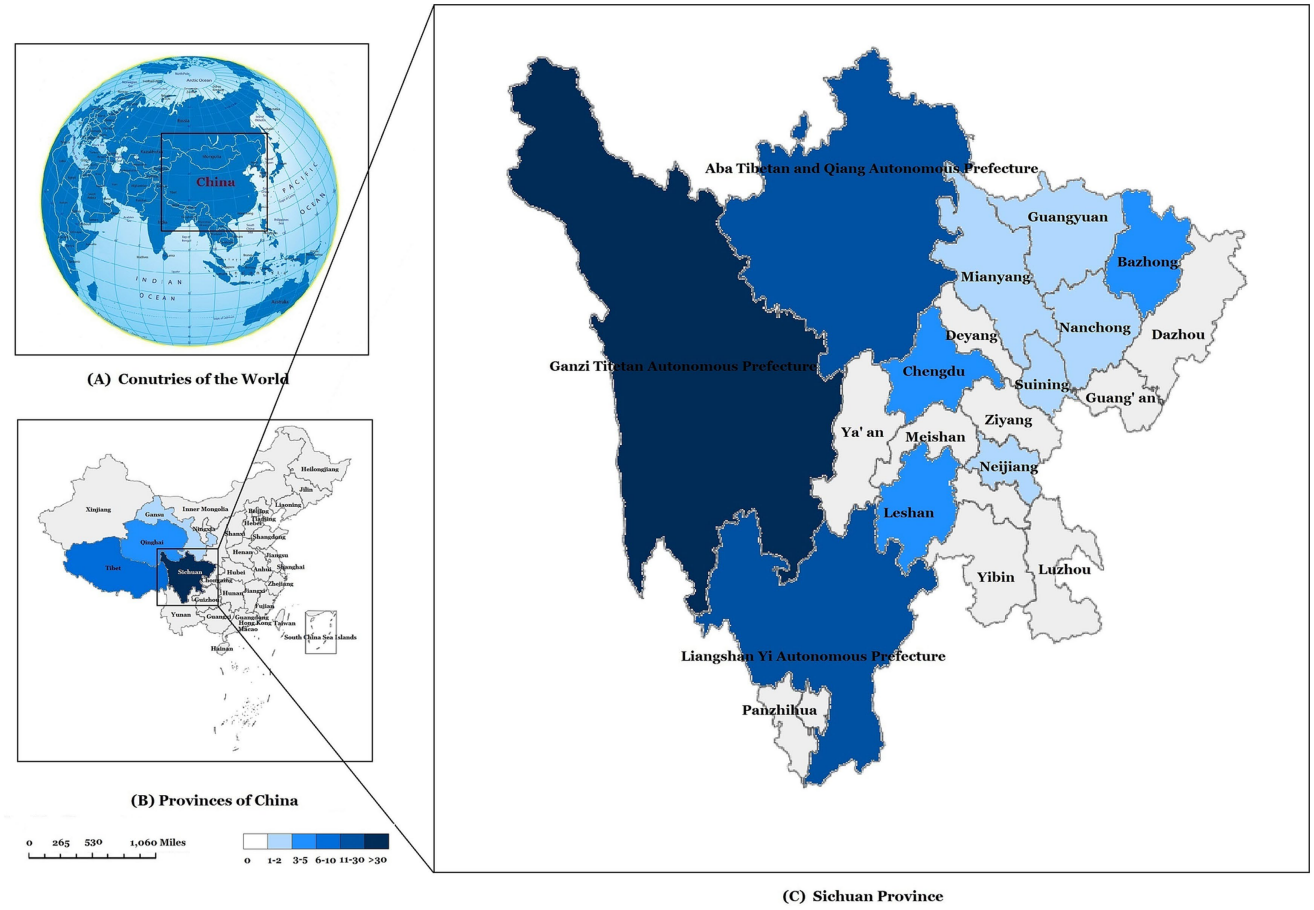
Geographical distribution of pediatric drug-resistant TB cases in our study. **(A)** Map of the study area. China location. **(B)** The geographical distribution of pediatric drug-resistant TB cases in China. **(C)** The geographical distribution of drug-resistant TB cases in Sichuan province.

Among 2,208 children with TB from 2017 to 2024 included in this study, the incidence rate of pediatric DR-TB increased from 0.42% in 2017 to 5.61% in 2024, with an average incidence rate of 4.08%, showing an upward trend year by year (*p* = 0.011) (Figure 4). The 90 pediatric DR-TB patients diagnosed and treated at PHCC between January 2017 and December 2024 were matched to a 1:100,000 digital map of China using Python 3.7. The geographical distribution map indicated that over the 8-year period, twelve of the pediatric DR-TB patients were from neighboring cities outside Sichuan Province [including Tibet (8 cases), Qinghai (3 cases), and Gansu (1 case)], while the remaining cases were from within Sichuan Province, primarily from the western Sichuan, Ganzi, Aba, and Liangshan minority areas, with the highest number of cases in the Ganzi Tibetan Autonomous Prefecture. The remaining cases were mainly distributed in Chengdu, Leshan, Bazhong, and other central urban areas (Figure 5).

## Discussion

In recent years, the increasing prevalence of DR-TB has added to the burden of TB in Southwest China, especially regarding DR-TB in children. Based on data from children with TB admitted to PHCC, which serves as the most representative centralized TB management unit in southwest China, over the past 8 years, this study analyzed the epidemiological and clinical characteristics of DR-TB in children and provides the first report on the special geographical distribution, epidemiological characteristics, and drug-resistant incidence trend of DR-TB in children in southwest China.

The incidence rate of TB in men is usually higher than that in women. According to WHO data, the incidence rate of TB in adult men is about twice that of adult women. The incidence rate of TB in children shows less difference between sexes, but is generally slightly higher in male than female children (World Health Organization, 2024). Due to the relatively limited data on pediatric DR-TB incidence, a study on DR-TB in children in Chongqing, China showed a slightly higher incidence in boys than girls (male-to-female ratio of 1.15:1) (Guo et al., 2016). In this study, however, the incidence rate among girls with DR-TB was significantly higher than that among boys, with a male-to-female ratio of 0.76:1, differing from the overall TB incidence trend. These findings align with a study on MDR-TB in children in Delhi, India that also reported higher prevalence in girls than boys (Arora et al., 2017). Furthermore, this study found particularly high proportions of MDR-TB among adolescent girls. Previous studies show that sputum smear-positive pediatric TB is more common in adolescent girls than boys of the same age (Bolursaz et al., 2016). This finding suggests a potential relationship between TB development and hormonal changes at menarche and adolescence (Comstock et al., 1974).

In this study, the peak incidence rate of DR-TB in children occurred in 2021 (7.17%), with an average incidence rate of 4.08% over the 8-year period. It is slightly lower than the studies in domestic places such as Shandong (18.9%) (Tao et al., 2017) and Shenyang (27.4%) (Sun et al., 2023). On the one hand, this difference may be due to certain geographical differences between the north and the south. On the other hand, it may also be because the groups of the above two studies are based on children under the age of 18, while the group of this study is based on children under the age of 14. Meanwhile, because this study included only discharged patients from PHCC over 8 years and not all outpatient cases, the case base may have some selection bias. Thus, the actual incidence of DR-TB in children in southwest China may be higher, though the hospital-based incidence showed an upward trend during these 8 years. According to data from West China Second Hospital of Sichuan University, the DR-TB rate in children was 10.4% from 2010 to 2014, with 76.1% being retreated cases (Liao et al., 2017). By contrast, this study found initial treatment resistance rates reaching 74.4% (RR-TB and MDR-TB accounted for 36.7 and 30.0%, respectively), while MDR-TB comprised only 16.7% of recurrent cases. These changes suggest that with increasing standardization of TB diagnosis and treatment along with medical advances, TB management has significantly improved. They also indicate that recent DR-TB transmission in children may result less from nonstandard treatment and more from direct infection through close contact with drug-resistant strains in households, communities, and schools. Therefore, screening and treatment for pediatric TB should be intensified in key areas, particularly through school-based screening in high-burden regions.

Our analysis of DR-TB epidemiology in southwest Chinese children confirmed serious DR-TB prevalence in Sichuan, Tibet, Qinghai, and neighboring regions, particularly in Ganzi, Aba, and Liangshan ethnic minority areas of Sichuan Province. These findings are consistent with our previous study on pediatric TB distribution in southwest China (Wang et al., 2024). Notably, Tibetan children accounted for 63.3% of pediatric DR-TB cases in this series. While Tibetan areas show high proportions of both pediatric TB and DR-TB cases, DR-TB is particularly overrepresented. This highlights relatively poor medical infrastructure in southwest Tibetan areas, where TB diagnosis and treatment may lack standardization. Geographic remoteness may also hinder consistent medication adherence and treatment completion, potentially increasing drug resistance risk. We recommend intensified TB screening and treatment programs in southwest China’s ethnic minority regions, especially Tibetan areas, with particular attention to treatment adherence.

This study has several limitations. First, it included only hospitalized pediatric TB patients treated at PHCC over 8 years, excluding outpatient cases, which may introduce selection bias. Second, among DST results, only 33 cases were culture-confirmed, while 57 relied on molecular diagnosis, limiting comprehensive drug resistance analysis across all phenotype-tested drugs.

In summary, this study addresses the data gap on pediatric DR-TB in southwest China and demonstrates the severe DR-TB and MDR-TB situation there, including unique geographical distribution, epidemiological features, and resistance trends. Our results underscore the urgent need to strengthen pediatric DR-TB control in southwest China, which affects both local child health and global TB elimination efforts.

## Data Availability

The original contributions presented in the study are included in the article/supplementary material, further inquiries can be directed to the corresponding author.
